# Supplementary material for: Circulating Tumor Cells Are Detectable and Independent of PSA and PSMA-PET Metrics in Localized High-Risk and Biochemically Recurrent Prostate Cancer
Source: medRxiv. 2025 Jul 15:2025.07.09.25331014. Preprint. [Version 2] doi: 10.1101/2025.07.09.25331014 (PMC12265774; doi:10.1101/2025.07.09.25331014)
Supplement: Supplement 1 — Supplementary Figure 1. Threshold determination, gating strategy, and assay validation for EpCAM and PSMA detection (A) Representative gating strategy used for the detection of EpCAM-positive circulating tumor cells (CTCs) by flow cytometry. From the nucleated viable cells, CD45-negative events were gated and further analyzed for EpCAM positivity. (B) Summary table establishing a CTC count threshold of ≥3 for positivity in the flow group. The cutoff was determined based on analysis of female healthy donors (HD), who served as negative controls. (C) Threshold determination for EpCAM and PSMA expression levels in the ddPCR group. CD45-depleted RNA from female healthy donors was used to establish background expression, and thresholds were calculated as mean + 2 standard deviations (2SD). (D) Sensitivity testing for EpCAM and PSMA detection using 22Rv1 prostate cancer cells as a positive control. This assay confirms the ability of ddPCR platform to reliably detect marker expression at known positive levels. [file media-1.pdf]

Figure S1

A] Gating strategy of Flow group for EPCAM positive CTCs

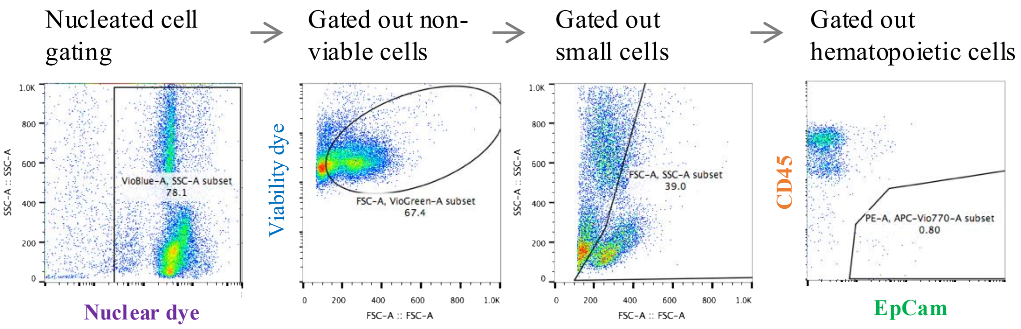

B) Flow group

Healthy Donor	EpCam+ Cell Count
HD1F	3
HD2F	3
HD3F	0
HD4F	0
HD5F	1
HD6F	0
HD7F	0
HD8F	1
HD9F	0
HD10F	0

C] ddPCR group

Samples	Target	Conc. (copies/μL)	Target	Conc. (copies/μL)
22Rv1	EPCAM	6,558.11	PSMA	4,870.48
HD1D	EPCAM	0.03	PSMA	0.00
HD2D	EPCAM	2.67	PSMA	0.00
HD3D	EPCAM	7.03	PSMA	0.00
HD4D	EPCAM	4.10	PSMA	0.00
HD5D	EPCAM	2.74	PSMA	0.00
HD6D	EPCAM	19.52	PSMA	0.00
HD7D	EPCAM	0.03	PSMA	8.59
HD8D	EPCAM	0.00	PSMA	1.75
HD9D	EPCAM	28.07	PSMA	3.94
HD10D	EPCAM	24.28	PSMA	5.42
NTC	EPCAM	0.09	PSMA	0.00

	EpCAM	PSMA
Mean	8.85	1.97
Standard deviation	10.84	3.04
Threshold (Mean + 2*Standard dev)	30.52	8.05

D] EpCAM and PSMA sensitivity testing using 22Rv1 cell line

RNA (pg)	EPCAM (copies/ul)
1000	3,505.86
200	702.67
40	173.06
8	22.41
4	10.14
2	6.38
1	2.45
0	0.00

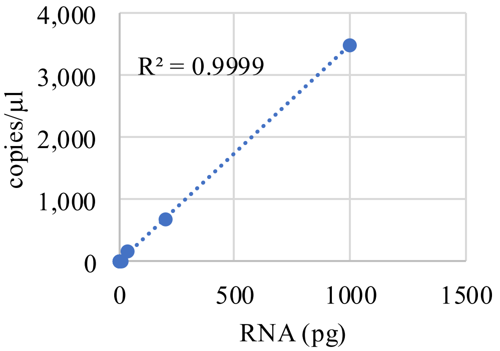

RNA (pg)	PSMA (copies/ul)
1000	12,478.73
200	3,670.80
40	860.58
8	121.35
4	55.74
0	0.00

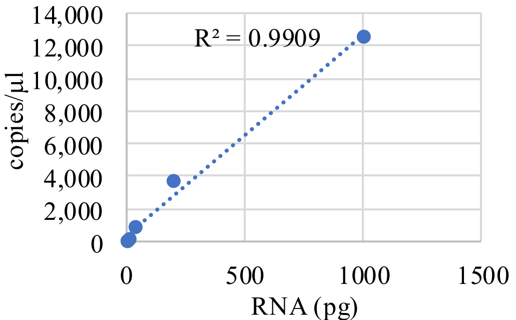

Without amplification		Copies/μl
Rv1_100ng	EPCAM	340.63
Rv1_100ng	PSMA	1,899.24
