# Supplementary material for: Circulating Tumor Cells Are Detectable and Independent of PSA and PSMA-PET Metrics in Localized High-Risk and Biochemically Recurrent Prostate Cancer
Source: medRxiv. 2025 Jul 15:2025.07.09.25331014. Preprint. [Version 2] doi: 10.1101/2025.07.09.25331014 (PMC12265774; doi:10.1101/2025.07.09.25331014)
Supplement: Supplement 2 — Table 1. Baseline clinical and demographic characteristics of patients in the Flow group. This table summarizes key clinical and demographic features of patients in the Flow group, including age, gender, disease status, and relevant laboratory parameters such as Gleason score and PSA levels at study entry. Table 2. Baseline clinical and demographic characteristics of patients in the ddPCR group. This table presents the key clinical and demographic characteristics of patients in the ddPCR group, including age, gender, disease status, and relevant laboratory parameters such as Gleason score and PSA levels at the time of study entry. [file media-2.pdf]

Table 1: Patient characteristics- Flow group

Characteristics		
Median age of combined cohorts (years old)		66.50 [48-85] [min-max]
High Risk (Cohort 1)		N= 14 (16.86%)
Biochemical recurrence (Cohort 2)		N= 68 (83.13%)
Clinical or pathological T stage at the day of blood drawn		T1c: N= 24 (Cohort 1 N= 10, Cohort 2 N= 14)<br>T2: N= 26 (Cohort 1 N= 1, Cohort 2 N= 25)<br>T3: N= 30 (Cohort 1 N= 3, Cohort 2 N= 27)<br>T4: N= 2 (Cohort 1 N= 0, Cohort 2 N= 2)
Median original Gleason score	Cohort 1	8 [6-10] [min-max]
	Cohort 2	7 [6-10] [min-max]
Median PSA level (ng/ml)	Cohort 1	13.01 [0.21- 925.9] [min-max]
	Cohort 2	1.96 [0.2-33.31] [min-max]
No treatment (Cohort 1)		N= 14
Pre-treatment (Cohort 2)		Prostatectomy N= 46<br>Radiation therapy N= 22
Median EpCAM CTC count	Cohort 1	2 [0-13] [min-max]
	Cohort 2	1 [0-25] [min-max]
Progression since date drawn to last available visit	Cohort 1	Yes (N= 4), No (N= 6), N/A (N= 4)
	Cohort 2	Yes (N= 27), No (N= 25), N/A (N= 16)

Table 2: Patient characteristics- ddPCR group

Characteristics		
Median age of combined cohorts (years old)		67 [51-81] [min-max]
High Risk (Cohort 1)		N= 17 (73.91%)
Biochemical recurrence (Cohort 2)		N= 6 (26.08%)
Clinical or pathological T stage at the day of blood drawn		T1c: N= 5 (Cohort 1 N= 4, Cohort 2 N= 1)<br>T2: N= 8 (Cohort 1 N= 4, Cohort 2 N= 4)<br>T3: N= 10 (Cohort 1 N= 9, Cohort 2 N= 1)<br>T4: N= 0 (Cohort 1 N= 0, Cohort 2 N= 0)
Median original Gleason score	Cohort 1	7 [6-9] [min-max]
	Cohort 2	8 [6-9] [min-max]
Median PSA level (ng/ml)	Cohort 1	14.0 [5.6-37.4] [min-max]
	Cohort 2	0.45 [0.3-4.8] [min-max]
No treatment (Cohort 1)		N= 17
Pre-treatment (Cohort 2)		Prostatectomy N= 5<br>Radiation therapy N= 1
Median EPCAM and PSMA expression (copies/µl)	Cohort 1	EPCAM: 35.7 [0-75.13] [min-max]<br>PSMA : 2.32 [0-37.85] [min-max]
	Cohort 2	EPCAM: 12.86 [0-51.93] [min-max]<br>PSMA: 2.995 [0-10.5] [min-max]
Progression since date drawn to last available visit	Cohort 1	Yes (N= 2), No (N= 10), N/A (N= 5)
	Cohort 2	Yes (N= 0), No (N= 4), N/A (N= 2)
